# Waterjet pulse lavage as a safe adjunct to video assisted retroperitoneal debridement in necrotising pancreatitis

**DOI:** 10.1007/s00464-024-11297-6

**Published:** 2024-10-04

**Authors:** Krishna Kotecha, John Yeh, Juanita N. Chui, Kevin Tree, Douglas Greer, Alex Boue, Tamara Gall, Siobhan McKay, Anubhav Mittal, Jaswinder S. Samra

**Affiliations:** 1 Department of Upper Gastrointestinal Surgery, Royal North Shore Hospital, Reserve Road, St Leonards, 2065 NSW Australia; 2 Northern Clinical School, University of Sydney, St Leonards, Australia; 3 School of Medicine, University of Notre Dame, Sydney, Australia; 4 Institute of Cancer and Genomic Science, University of Birmingham, Edgbaston, Birmingham, B15 2TT UK

**Keywords:** Pancreatitis, Pancreas, Necrosectomy

## Abstract

**Background:**

Minimally invasive surgical necrosectomy plays an important role in the management of infected pancreatic necrosis, with a goal of removing debris and debriding necrotic tissue. Pulse lavage is designed to simultaneously hydrostatically debride and remove the infected necrotic tissue with suction. It is also able to remove significant amounts of debris without traumatic manipulation of the necrotic tissue which may be adherent to surrounding tissue and can result in injury.

**Methods and results:**

The surgical technique of utilising a waterjet pulse lavage device during the minimally invasive necrosectomy is detailed. Sixteen patients being managed via a step-up approach underwent endoscopic necrosectomy via a radiologically placed drain tract. All sixteen patients were successfully managed endoscopically without conversion to open necrosectomy, and survived their admission. There were no complications associated with the use of the waterjet pulse lavage.

**Conclusion:**

Waterjet pulse lavage is a useful adjunct in minimally invasive necrosectomy, which reduces the length of the necrosectomy procedure, and facilitates removal of necrotic tissue while minimising the risk of traumatising healthy tissue.

**Supplementary Information:**

The online version contains supplementary material available at 10.1007/s00464-024-11297-6.

Necrotizing pancreatitis (NP), which can occur in up to 20% of patients with acute pancreatitis (AP), is a life-threatening condition associated with organ failure and a multitude of ischaemia driven complications [1]. If the necrotic tissue becomes secondarily infected (typically in the second to third week after initial presentation), the mortality rates increases from approximately 12% in the absence of infection to 40–70% [2]. Therefore, pancreatic necrosectomy plays an important role in reducing the circulating inflammatory and infectious burden. Historically this was achieved by open necrosectomy; to reduce the morbidity associated with this procedure, several less invasive approaches have been described. These include percutaneous drainage, endoscopic transluminal necrosectomy (ETN), video assisted retroperitoneal debridement (VARD), grouped under the term minimally invasive pancreatic necrosectomy (MIN).

Patients undergoing MIN are known to have reduced incidence of post-operative organ failure and a lower likelihood of ICU admission when compared to historical controls who underwent open surgery. These findings were validated by the randomised PANTER trial, which confirmed lower rates of morbidity and mortality in patients managed by a ‘step-up’ approach [2], where percutaneous drainage is followed by progressively more invasive approaches based on pre-defined parameters. When compared to open necrosectomy, VARD and other minimally invasive surgical techniques consistently show lower rates of morbidity, organ failure, and mortality [3], and are therefore preferable in the management of NP.

VARD often performed laparoscopically via a pre-existing retroperitoneal drain tract, and involves the use of grasping forceps [4] to pull necrotic tissue away from the healthy pancreas. This method has been known to lead to bleeding (from the splenic artery and its branches), and injury to adjacent organs (e.g. colon or spleen) [4], often necessitating conversion to an open operation. Even under direct vision, it is difficult to predict the location of these structures in relation to overlying necrotic tissue that must be removed. A useful augmentation to the VARD is the waterjet pulse lavage device (National Surgical Corporation, Caringbah NSW Australia); a single use disposable system consisting of a hand piece, waste pipe, irrigation pipe, and battery pack (Fig. 1B). The device is equipped with a standard long and short nozzle, and it is the long nozzle (30 cm) that augments VARD. The device allows simultaneous irrigation and suction from the tip of the nozzle. The irrigation pressure at the tip is adjustable—with a low setting of pressure 9psi/0.6205 bar/0.062 mPA and flow rate < 700 ml/min, or high pressure setting at 15psi/1.034 bar/0.1034 bar and flow rate > 1000 ml/min. This allows for the rapid debridement and removal of necrotic tissue and infected debris. Using the pulse lavage device avoids the inadvertent shearing of healthy tissue with laparoscopic graspers, reducing morbidity and mortality.

**Fig. 1:**
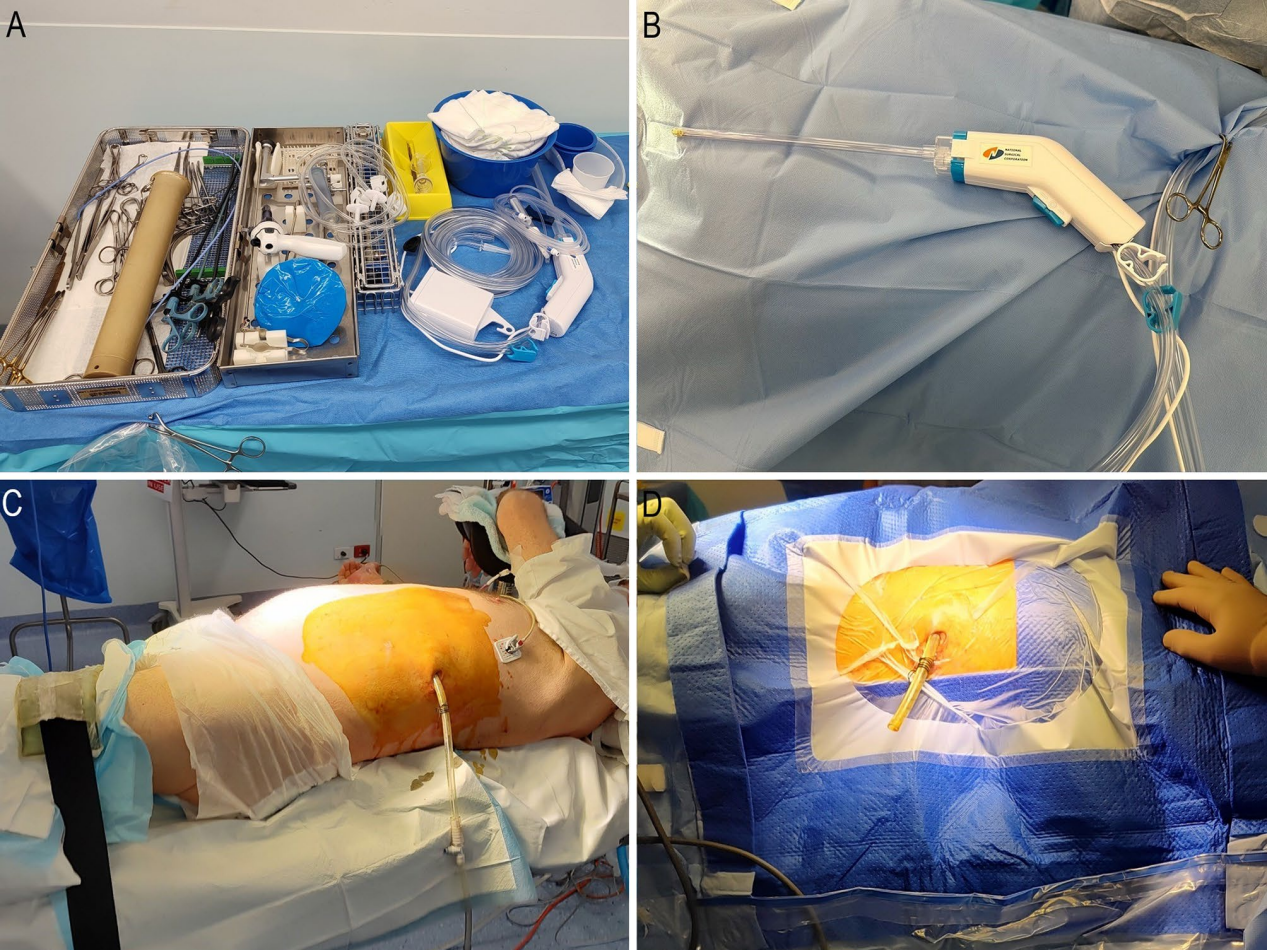
**A** Minimally invasive necrosectomy set-up. **B** Waterjet pulse lavage equipment. **C** Patient positioned with a wedge under the left side. **D** Prepped and draped patient with drain cut short

## Materials and methods

Patients were managed in a multidisciplinary fashion with ICU, radiology, and gastroenterology. The diagnosis of infected necrosis was made based on any of the following: presence of gas in peripancreatic fluid on imaging; positive blood cultures with typical organisms with or without features of sepsis; or confirmed infection on diagnostic aspirates. Timing of VARD was determined on a case by case basis, but was often 18–21 days after initial presentation.

Necrotic collections were managed via a step-up approach [5];

1. A pigtail catheter (Cook Medical, Bloomington IN, USA) was placed percutaneously in the peripancreatic collection under the guidance of CT. Minimal drain size was 12-Fr, and each collection was drained separately. The preferred access route was through the left retroperitoneum.
2. For irrigation, the drain was placed on constant lavage of normal saline running at 100 mL/hour.
3. If the position of the drains was inadequate, or further remnant collections were amenable to drainage, then the percutaneous drainage procedure was repeated
4. Step up to VARD occurred if there was no clinical improvement based on clinical status, systemic inflammatory markers, or on progress CT imaging after 72 h as per the PANTER criteria

*Ethics approval for this project was granted by the district ethics board* (NSLHD HREC 2021/ETH00479).

### Operative technique (see attached video)

Imaging is reviewed by the operating surgeon before commencing the procedure to ascertain:

- Distance from skin to cavity
- Direction of travel of the drain
- Proximity between the drain and any adjacent structures (e.g. spleen, colon, stomach, vessels)

### Patient positioning

Patients are positioned supine with a wedge under the left shoulder and hip aiming for 30–45 degrees tilt of left side up (Fig. 1C and D). The left arm is placed in an arm gutter and a pillow placed between the legs. The aim of positioning is to have the left posterior axillary line and flank exposed, providing adequate space below the drain insertion site to allow the operator to move the scope comfortably in all directions and not be limited by the bed.

### Surgical technique

**Fig. 2:**
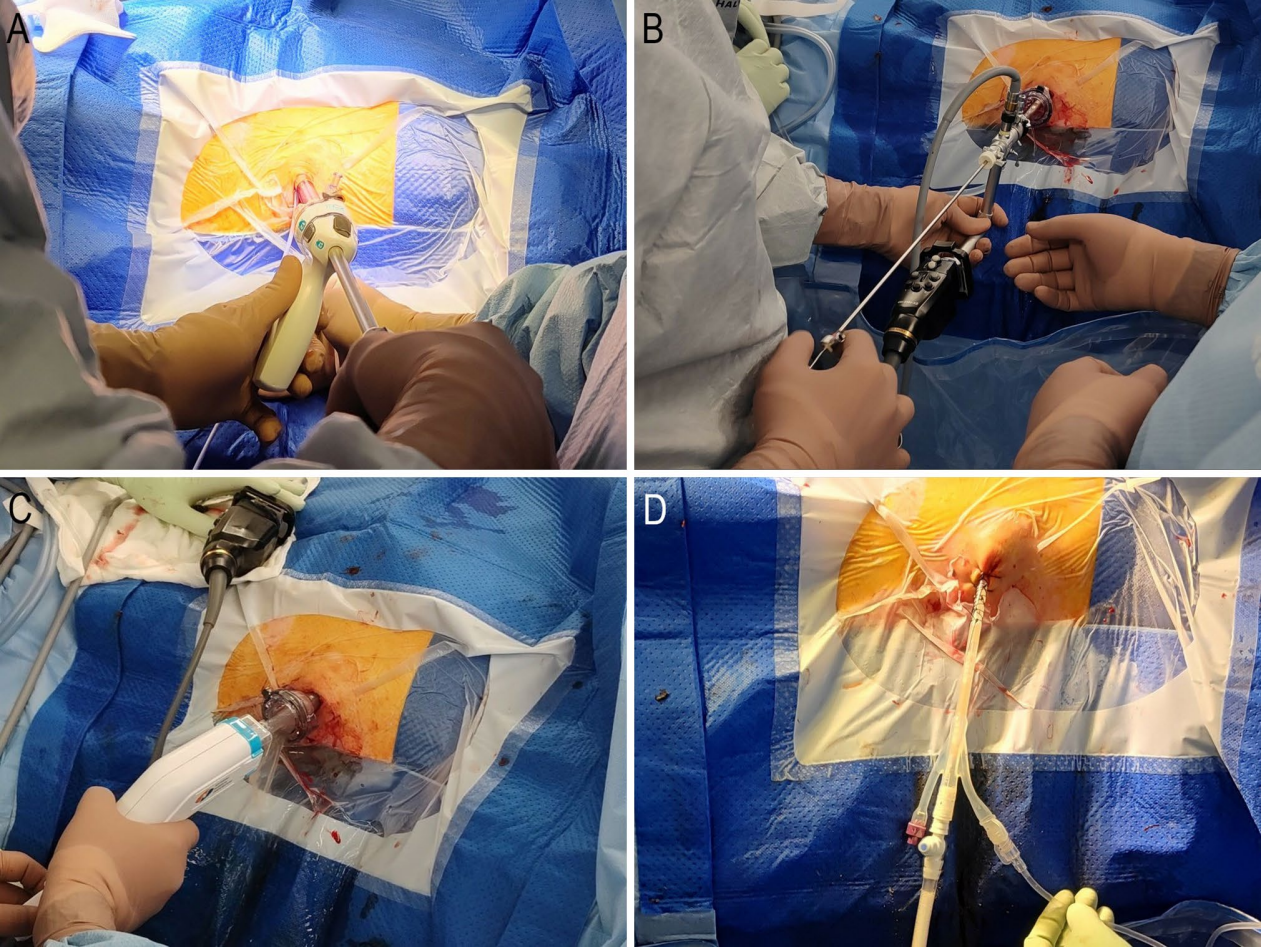
**A** Optiview entry under vision into the drain tract. **B** A nephroscope can be used to facilitate graspers and manual debridement following loosening of the necrotic tissue with the pulse lavage. **C** Insertion of pulse lavage device with suction-irrigation. **D** Insertion of three-way urinary catheter as a drain, cut to size, and with continuous irrigation

**Fig. 3:**
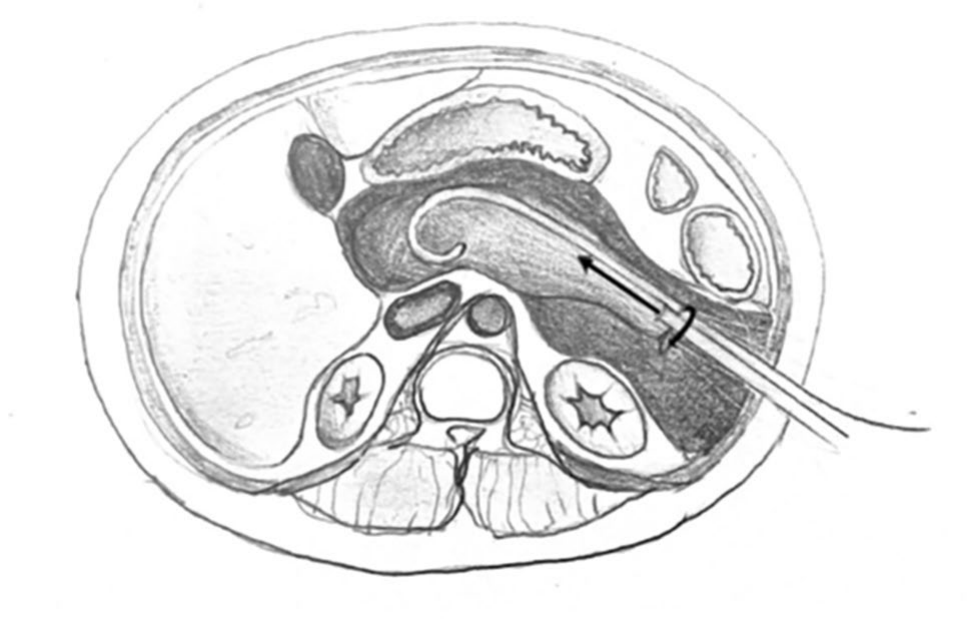
The pulse lavage instrument is advanced longitudinally only in the direction of the drain tract, and rotated upon its axis

An incision is made at the site of the drain, and optical entry is achieved with a 12 mm OptiView trocar (Ethicon, Somerville NJ, USA) and a 0° 10 mm rigid endoscope, allowing access to the retroperitoneum without the need for open cutdown (Fig. 2A). The port is advanced along the existing pigtail drain, ensuring continuous visualisation of the drain until the cavity is reached. The distance between the skin and necrotic tissue is noted using the rigid endoscope. Laparoscopic suction is utilised to facilitate drainage of any pus. Subsequently, the 0° camera is exchanged for a nephroscope to allow the surgeon to define the size, shape, and longitudinal orientation of the cavity and collections. The nephroscope is then replaced with the long nozzle of the pulse lavage device, which is inserted to the predetermined distance (Fig. 2C). Alternatively, a choledochoscope can be inserted in the port to allow simultaneous placement of the pulse lavage device, facilitating the procedure under direct vision. The pulse lavage is activated in short bursts (5–10 s) to prevent pressure build up in the retroperitoneum. The pulse lavage device is advanced longitudinally in the direction of the established cavity and rotated 360 degrees around its axis (Fig. 3). The primary goal is to prevent any direct blunt trauma to the intracavity tissue. The following principles are observed at all time.

The instrument:

- must not be pushed against resistance
- must not be moved while active, as the suction can damage healthy tissue
- must only be moved along the drain tract axis—i.e. move only to ‘push and pull’ or ‘twist to rotate’ along the established tract

Pulse lavage can continue until the effluent in the suction tubing is clear. Atraumatic graspers can then be used to remove any loosely adherent necrotic tissue (Fig. 2B) which has separated from the healthy granulating tissue. In case of minor ooze from the granulating tissue, temporary packing can be used to stop the bleeding. If a bleeding vessel is seen, a clip applicator or harmonic scalpel can be used for haemostasis. In the rare event where bleeding cannot be controlled, packing of the retroperitoneal cavity can be performed as a bridge to angioembolisation.

Patients often require multiple debridements; it is therefore reasonable to set a time limit (e.g. one hour) per debridement session; This has the advantage of:

- Minimising fluid and electrolyte shifts
- Allowing borderline tissues an opportunity to declare themselves as either necrotic or viable
- Avoiding surgeon fatigue

At completion, a large drain is inserted which must allow continuous irrigation. A readily available 3-way catheter is placed within the cavity, with a stoma bag placed over the drain/operative site to manage any leakage from around the drain (Fig. 2D). Normal saline irrigation is then commenced at 100 ml/hr for 24 h.

### Post-operative management

If required, patients were managed in the Intensive Care or High-Dependency Unit for 24 h for close observation. Progress imaging (CT) is obtained at 5–7 days post procedure (or sooner if clinical deterioration dictates) and repeat VARD was performed as required. Intravenous antibiotics were continued with infectious diseases specialist involvement, and dietetics involvement for nutritional optimisation.

## Results

Between January 2017 and March 2024, sixteen patients underwent VARD with pulse lavage (Characteristics listed in Table 1 and Table 2).

**Table 1:** Patient demographics, treatment information, and outcomes

Median age, years (range)	61 (32–81)
Sex, number (%)	
Male	10 (62.5%)
Female	6 (37.5%)
ASA, number (%)	
III	8 (50%)
IV	8 (50%)
Median BMI (range)	26.9 (20.2–35.4)
Current or ex-smoker, number (%)	
Yes	6 (37.5%)
No	10 (62.5%)
Aetiology of pancreatitis, number (%)	
Gallstones	8 (50.0%)
Alcohol	2 (12.5%)
Idiopathic	3 (18.8%)
Triglycerides	1 (6.3%)
Hypercalcemia	1 (6.3%)
Post-ERCP	1 (6.3%)
Median number of percutaneous drains prior to MIN (range)	1.5 (1–2)
Median number of MIN (range)	2 (1–7)
Median number of pancreas CT scans (range)	17 (6–32)
Clavien-Dindo complications, number (%)	
II	3 (18.8%)
IIIa	6 (37.5%)
IIIb	1 (6.3%)
IVa	3 (18.8%)
IVb	2 (12.5%)
V	1 (6.3%)
Median length of stay, days (range)	
Total	100 (63–163)
Before first MIN	42 (18–88)
After first MIN	55 (23–136)
Survival, number (%)	
30-day	16 (100%)
90-day	16 (100%)

**Table 2:** Peri-operative variables relating to severity of disease process

ICU admission, number (%)	
Pre-operative	13 (81.3%)
Post-operative	12 (75.0%)
Median CRP, mg/L (range)	
Peak	381 (191–595)
ICU admission	200 (50–349)
Immediately prior to MIN	187 (26–382)
Immediately pre-operative variables	
Median APACHE II score (range) Median temperature, Celsius (range)	11.5 (1–31) 36.8 (36.5–39.2)
Median MAP, mmHg (range)	76.5 (55–117)
Median heart rate, BPM (range)	7.40 (7.12–7.49)
Median respiratory rate, BPM (range)	101 (63–140)
Acute kidney injury, number (%)	7 (43.8%)
Median haematocrit (range)	0.323 (0.231–0.516)
Median white cell count, × 10^9^/L (range)	15.1 (5.8–39.6)
Requirements any time prior to MIN, number (%)	
Ventilation	6 (37.5%)
Blood pressure support	7 (43.8%)
Dialysis*	2 (12.5%)
Requirements at time of MIN, number (%)	
Ventilation	6 (37.5%)
Blood pressure support	4 (25.0%)
Dialysis*	2 (12.5%)

^*^one patient already on dialysis for chronic renal failure

Prior to the first VARD, half of the patients had an ASA of 4 and the median APACHE II score was 11.5 (range 1–31). Thirteen patients (81.3%) required ICU management pre-operatively, and twelve patients (75.0%) required ICU care post-operatively. Patients were admitted for a median of 42 days (range 18–88 days) before their first VARD. All patients had CT-guided retroperitoneal drainage prior to VARD (median of 1.5 procedures) prior to proceeding to VARD.

No patients had complications from their VARD. However, all patients had further complications of pancreatitis. These complication are detailed in Supplementary Table 1, and included CD 3a (pleural effusion requiring chest drain; intraabdominal collection requiring further IR guided drainage; duodenal fistula managed with IR drainage; pseudoaneurysm of middle colic artery requiring embolisation); CD 3b (patient requiring endoscopic cystgastrostomy for further collections); CD 4a (abdominal compartment syndrome necessitating laparotomy; renal failure requiring dialysis); and CD 4b (ventilator associated pneumonia with PEA arrest and heart failure; massive bleed from splenic artery requiring massive transfusion and splenic artery embolisation). The patient who suffered a PEA arrest survived his admission and died 13 months later in the community. There was only one in-hospital mortality, which was a patient with progressive multi-organ failure after a nearly 6-month long admission.

## Discussion

Pancreatic necrosis is a dangerous complication of acute pancreatitis [6], and if infected, mortality can be as high as 70% [7]. Removal of all necrotic tissue, and the evacuation of infected material is necessary to reduce the systemic inflammatory burden [4]. Open necrosectomy is associated with post-operative complication rates of 34–95% [8] and mortality rates of 11–39% [9], and is associated with long-term adverse outcomes including cutaneous fistulae, endocrine and exocrine insufficiency, and abdominal wall hernia. The short- and long-term advantages of a step-up approach using MIN is now well established. [10]

Minimally invasive methods can be classified by access route (peritoneal, retroperitoneal, transoral), or by method used for visualisation (laparoscopic, rigid nephroscopic, flexible endoscopic, endoscopic ultrasound guided). [4]

The step-up approach includes percutaneous drainage or endoscopic (transgastric drainage), before proceeding to minimally invasive retroperitoneal necrosectomy, and then open necrosectomy. With endoscopic necrosectomy, there remains a substantial risk of adverse events and mortality, including bleeding, introduction of infection into the necrosis, exocrine and endocrine insufficiency, with reported mortality of up to 7.5% and morbidity rate of 14–26% [5]. However, where possible, endoscopic (transgastric) drainage is favoured in our centre– recent meta-analysis [11] demonstrates potentially reduced rates of multiple organ failure, visceral perforations, and fistulae (both enterocutaneous and pancreatic) and lower mean hospital stay with endoscopic debridement. Additionally, the Dutch exTENSION trial [12] shows that patients undergoing endoscopic debridement require fewer interventions than those undergoing surgery. This is reflected in the findings of Ramai et al [13]; when compared to surgery and percutaneous drainage, patients who undergo endoscopic necrosectomy have significantly lower risk of inpatient mortality, adverse events, length of stay, and cost [14]. We also observe that endoscopic debridement and internal drainage results in improved quality of life and patient tolerance, compared to external drainage. Patients with necrotising pancreatitis are discussed at a multidisciplinary board meeting, consisting of HPB surgeons, gastroenterologists, interventional radiologists, nutritionists, as well as others. Endoscopic necrosectomy remains the preferred approach to management of peripancreatic necrosis in our institution. These patients were deemed inappropriate for endoscopic management due to a variety of factors, including the location of the necrosis (and relation to splenic vessels), distance from stomach lumen, size and density of necrosis, number of necrotic collections, and endoscopist preference. As seen with this study cohort, patients who are not suitable for endoscopic debridement receive percutaneous drainage, and can then undergo pulse lavage necrosectomy.

For percutaneously placed drains, a retroperitoneal approach is favoured to avoid peritoneal contamination and enteric injury [15]. The subsequent control of infected necrosis means that open necrosectomy may be deferred or avoided. This approach induces less stress than open surgery in already critically ill patients [16].

The use of the Optiview trocar in this approach avoids the need for a subcostal incision and fascial cutdown, as described in other methods [17, 18]. The continuous pulse lavage, which allows continuous irrigation and suction of necrotic debris and tissue, enhances debridement under vision when compared to use of graspers to remove necrotic tissue, which may be time-consuming and has the potential to cause injury to underlying tissue, particularly the splenic vessels and their branches. The pulse lavage can more gently, and efficiently, debride necrotic tissue, as is commonly used in soft tissue infections. Pulsed waterjet dissection in a swine liver model has demonstrated that the pressure required to dissect healthy liver parenchyma is 1.41mPA (14 bar), and the pressure required to dissect the hepatic vein is 8.66 mPa (86.6 bar) [19]. The pulsejet pressure in this series is set a maximum pressure of 1 Bar, which safely fragments necrotic pancreatic tissue without damage to healthy tissue. The mechanism of tissue fragmentation is irrigation alone; there is no direct contact of pancreatic tissue against the device or mechanical manipulation of necrotic tissue. If the waterjet is positioned a short distance away from the necrotic tissue, an additional margin for safety can be achieved; previous waterjet dissection data suggests that water pressure drops by 50% at 3 mm from the nozzle tip and by 90% at 3.5 mm from the nozzle tip [20].

An encountered disadvantage is the lack of specialised device to perform the procedure. Use of a 10 mm nephroscope or choledochoscope allows visualisation of the necrotic tissue and cavity; however, the pulse lavage nozzle cannot be placed simultaneously through the port. A choledochoscope, with its small calibre, can be used to perform the waterjet debridement under vision; however, it is limited in the working devices it can accommodate and in its irrigation rate. A specialised device with pulse lavage and camera should be investigated and developed further. Our overall experience has been that the pulse lavage is safe to use in this setting as a method of VARD, and is a useful adjunct to assisting MIN.

## Conclusion

The waterjet pulse lavage is a useful adjunct to video assisted retroperitoneal drainage for necrotising pancreatitis, and facilitates safe tissue debridement.

## Supplementary Information

Below is the link to the electronic supplementary material.

Supplementary file1 (DOCX 18 kb)
