# Supplementary material for: Waterjet pulse lavage as a safe adjunct to video assisted retroperitoneal debridement in necrotising pancreatitis
Source: Surg Endosc. 2024 Oct 4;38(11):6973–9. doi: 10.1007/s00464-024-11297-6 (PMC11525386; doi:10.1007/s00464-024-11297-6)
Supplement: Supplementary file 1 — Supplementary file1 (DOCX 18 kb) [file 464_2024_11297_MOESM1_ESM.docx]

Supplementary information

**Table 1** Disease course of each patient, including severity of pancreatitis (APACHE score)

| Patient | **AGE** | **SEX** | **Number of VARD** | **Comorbidites** | **APACHE II score immediately prior to 1st VARD** | Clavein Dindo | Post VARD disease course | **Complication related to VARD** | **Overall LOS (days)** |
| --- | --- | --- | --- | --- | --- | --- | --- | --- | --- |
| 1 | 32 | Male | 3 | hypertension, GORD, epilepsy | 1 | 3a | Gram negative bacteraemia. Further peripancreatic collections requiring percutaneous drainage. | No | 87 |
| 2 | 75 | Female | 2 | hypertension | 16 | 3a | duodenal fistula managed with radiologically inserted drains. atrial fibrillation, fluid overload including pleural effusions, gram positive bactaeraemia, upper limb superficial vein thrombosis – all managed medically. | No | 105 |
| 3 | 37 | Male | 3 | Type 2 Diabetes | 11 | 2 | Pulmonary embolus requiring anticoagulation. Line associated internal jugular vein thrombus requiring anticoagulation | No | 74 |
| 4 | 53 | Male | 2 | fatty liver disease, GORD | 5 | 2 | Lower limb deep vein thrombosis – anticoagulation. | No | 146 |
| 5 | 81 | Male | 2 | Atrial Fibrillation, hypertension, hypercholesterolemia, previous stroke, GORD | 12 | 3a | Transfusion requirement post VARD, with no bleeding demonstrated on CT angiogram. fluid overload, new atrial firbillation. pleueral effusion requiring chest drain | No | 72 |
| 6 | 46 | Male | 7 | nil | 13 | 2 | Fluid shift associated acute kidney injury | No | 163 |
| 7 | 78 | Male | 2 | Type 2 Diabetes, hypertension, hypercholesterolemia, hypothyroidism, | 31 | 4b | ventilatory associated pneumonia, PEA arrest requiring resuscitation | No | 161 |
| 8 | 77 | Female | 3 | Hypothyroidism, hypertension, hypercholesterolaemia, previous peptic ulcer disease, diverticular disease, depression | 21 | 4a | pseudoaneurysm of middle colic artery near walled off necrosis requiring angioebolisation. Gram negative bacteremia, intra-abdominal collections & renal abscess managed with antibiotics. COVID19 infection. New diabetes (type 3c). | No | 156 |
| 9 | 56 | Female | 1 | none | 8 | 3a | splanchnic vein (portal vein, SMV, IMV) thrombus – commenced on anticoagulation. intrabdominal collection requiring radiological drainage, colonic fistula to collection managed conservatively | No | 63 |
| 10 | 66 | Female | 4 | depression, hypercholesterolemia, hypothyroidism | 13 | 3b | Intrabdominal collections requiring percutaneous drainage. Further walled off necrosis amenable to endoscopic debridement- EUS cystgastrostomy. | No | 134 |
| 11 | 35 | Male | 2 | end stage renal failure secondary to hypertensive nephropathy, macroangiopathic haemolytic anaemia, hypertension, OSA | 14 | 3a | splenic artery pseudoaneurysms requiring transfusion and angioembolisation, gram negative bacteramia, intrabdominal collections requiring radiologic drainage | No | 161 |
| 12 | 53 | Male | 6 | nil | 4 | 4a | Gram negative bacteraemia, needing ICU for BP support | No | 93 |
| 13 | 71 | Female | 2 | Hypertension, hypercholesterolemia, paroxysmal atrial fibrillation, appendectomy, hysterectomy | 5 | 4a | Intrabdominal collections requiring radiologic drainage. Post-procedure to ICU for BP and ventilatory support | No | 81 |
| 14 | 68 | Male | 2 | Obstructive sleep apnoea, type 2 diabetes | 9 | 3a | GDA pseudoaneurysm requiring angioembolisations, radiologic drainage of intr-abdominal collections | No | 86 |
| 15 | 64 | Male | 3 | nil | 21 | 4a | splenic artery branch aneurysm and bleed requiring angioembolisation, laparotomy for abdominal compartment syndrome | No | 91 |
| 16 | 33 | Male | 2 | nil | 27 | 4b | renal failure requiring dialysis, ventilatory support | No | 95 |
|  |  |  |  |  |  |  |  |  |  |
